# Chronic stress elicits sex‐specific mitochondrial respiratory functional changes in the rat heart

**DOI:** 10.14814/phy2.70371

**Published:** 2025-05-12

**Authors:** Caitlin P. Odendaal‐Gambrell, Cassidy O'Brien, Megan Cairns, Gerald J. Maarman, Danzil E. Joseph, Carine Smith, Fanie Rautenbach, Jeanine L. Marnewick, M. Faadiel Essop

**Affiliations:** ^1^ Centre for Cardiometabolic Research in Africa (CARMA), Division of Medical Physiology, Biomedical Research Institute, Faculty of Medicine and Health Sciences Stellenbosch University Cape Town South Africa; ^2^ Centre for Cardiometabolic Research in Africa (CARMA), Department of Physiological Sciences, Faculty of Science Stellenbosch University Stellenbosch South Africa; ^3^ Experimental Medicine Unit, Department of Medicine, Faculty of Medicine and Health Sciences Stellenbosch University Stellenbosch South Africa; ^4^ Applied Microbial and Health Biotechnology Institute Cape Peninsula University of Technology Cape Town South Africa

**Keywords:** cardiovascular diseases, chronic restraint stress, chronic stress, heart, mitochondrial respiration, oxidative stress

## Abstract

Although chronic psychosocial stress is linked to cardiovascular diseases, the underlying mechanisms remain elusive. For this study, we focused on the mitochondrion as a putative mediator of stress‐related cardiac pathologies in a sex‐dependent manner. Male and female Wistar rats were subjected to chronic stress for 4 weeks (mimicking an anxious phenotype) versus matched controls. Cardiac redox status, mitochondrial respiration parameters, and expression levels of proteins involved in mitochondrial oxidative phosphorylation, dynamics, and biogenesis were evaluated. Despite limited changes in behavior and circulating stress hormones (both sexes), stressed males exhibited altered cardiac oxidative phosphorylation via β‐oxidation‐ and glucose oxidation‐linked respiratory pathways together with increased myocardial antioxidant capacity and decreased lipid peroxidation. Conversely, stressed females exhibited a protective and resilient phenotype by displaying augmented levels of major mitochondrial respiratory complexes (complex I, III, and ATP synthase) and a fusion marker (mitofusin‐2 [Mfn2]), together with attenuated expression of a fission marker (dynamin‐related protein‐1 [Drp1]) despite decreased estradiol levels. In contrast, stressed males displayed increased cardiac ATP synthase levels together with diminished peroxisome proliferator‐activated receptor‐gamma coactivator‐1‐alpha (PGC‐1α) expression versus controls. These findings indicate that male mitochondria are more prone to stress‐related functional changes, while females exhibited a more protective and resilient phenotype.

## 1 INTRODUCTION

Chronic stress is a major risk factor for the development and progression of cardiovascular diseases (CVD) (Munir & Du Toit, 2024; Osborne et al., 2020). The risk for CVD is closely linked with and exacerbated by modern‐day stressors, including work‐related stress, gender‐based violence, job insecurity, and the recent COVID‐19 pandemic. Chronic stress typically occurs due to a sustained stressor(s) or due to an acute event that continues to elicit a stress response over a prolonged period (Chu et al., 2022; Picard et al., 2018; Wittmann et al., 2019). This results in the activation of the hypothalamic–pituitary–adrenal (HPA) pathway. In this process, corticotropin‐releasing hormone (CRH) is released from the hypothalamus into the hypophysial portal system to ultimately promote the synthesis and release of adrenocorticotropic hormone (ACTH) from the anterior of the pituitary gland (Kaltsas et al., 2011). ACTH targets the adrenal cortex of the adrenal gland to produce glucocorticoids, a group of corticosteroids that are widely referred to as “stress hormones” (Burford et al., 2017; Godoy et al., 2018). Cortisol (corticosterone in rats) is the primary glucocorticoid and fundamental effector of HPA activation and is therefore employed as a well‐known biomarker of chronic stress (Spencer & Deak, 2017). The HPA pathway functions as a survival mechanism through the stimulation of various stress mediators to restore homeostasis (Steptoe et al., 2011). However, overstimulation of such mediators can lead to a maladaptive stress response that may potentiate numerous pathologies such as CVD (Chu et al., 2022; Picard et al., 2018).

Although various behavioral, lifestyle, and dietary factors contribute to such disease progression, the underlying physiological mechanisms driving stress‐related cardiovascular complications remain relatively unclear. While several candidates are implicated as putative mechanistic agents, the mitochondrion is emerging as an interesting mediator in this context. Here, intracellular energy required for homeostatic adaptation during the stress response is produced by a finely tuned orchestration of various processes that encompass oxidative phosphorylation (OXPHOS) together with the modulation of expression levels and activity of respiratory complexes, mitochondrial dynamics, and mitochondrial biogenesis (Picard et al., 2018). This is particularly relevant for the heart as its sustained contractile function and significant energy demands require a relatively high abundance of healthy cardiac mitochondria during stressful conditions (Murphy et al., 2016). However, the mitochondrion is paradoxical in nature and can trigger both beneficial and detrimental effects on cardiac function. Reactive oxygen species (ROS) are metabolic intermediates produced as natural by‐products of the electron transfer system (ETS) that are maintained at relatively low levels by various intracellular antioxidants (Picard et al., 2018). This allows ROS to play key roles in terms of intracellular signaling and gene expression pathways to help ensure cardiac cellular homeostasis (Nolfi‐Donegan et al., 2020). However, stressful conditions may result in relatively high ROS levels that can supersede intracellular antioxidant defense systems and culminate in oxidative stress, a well‐described mediator of CVD (Pizzino et al., 2017; Rahal et al., 2014). While an oxidative state may arise from various intracellular sources, the mitochondrion is a key driver (Dubois‐Deruy et al., 2020). This also places the mitochondrion at risk for oxidative stress‐induced damage and dysfunction. However, mitochondria are highly dynamic organelles that continuously undergo bioenergetic and fission‐fusion events to maintain their quality, abundance, morphology, and function (Ren et al., 2020).

Although previous research reported on the effects of chronic stress on mitochondrial function in animal models, there is a marked paucity of studies investigating the effects of chronic psychosocial stress in both males and females (Chiba et al., 2012; García‐Fernández et al., 2012; Kambe & Miyata, 2015; Liu et al., 2004; Liu & Zhou, 2012; Madrigal et al., 2001). As women are twice as likely to suffer from severe stress, anxiety, and depression compared to men, this emphasizes the need and clinical relevance for stress research to include both sexes (Issler & Nestler, 2018; Remes et al., 2016). Considering this, the current preclinical study aimed to assess the effects of chronic stress on cardiac mitochondria in a sex‐dependent fashion. Here, we hypothesized that chronic stress induces oxidative stress via a maladaptive stress response that contributes to altered cardiac mitochondrial respiratory function, dynamics, and biogenesis in a sex‐dependent manner.

## 2 MATERIALS AND METHODS

### 2.1 Animals

A total of thirty‐two male (*n* = 16) and female (*n* = 16) Wistar rats (7 weeks old) were acquired from and housed at the Tygerberg Animal Facility (Faculty of Medicine and Health Sciences, Stellenbosch University) in standard rat cages. Groups of four per cage (all assigned to the same experimental group) were housed in a temperature (22–24°C) and humidity‐controlled (40%–60%) room with ad libitum access to water and standard rodent chow (Rodent Breeder Customized Laboratory Animal Diet; #LAB/RB2005; NutritionHUB (Pty) Ltd.) except during experimental interventions. This soundproofed room operated on a 12‐h light–dark cycle (lights on from 7:00 a.m. to 7:00 p.m.) with limited personnel access. Here, the rats underwent a 2.5‐week habituation period with frequent daily handling, allowing them to acclimatize to their environment and handlers. Individual welfare monitoring of all animals took place throughout the experimentation period. Ethical clearance was granted by the Animal Care and Use Research Ethics Committee of Stellenbosch University (#ACU‐2022‐19,400), and all handling and treatment of the rats aligned with the accepted standards for the use of animals in research and teaching as reflected in the South African National Standards 10,386:2008.

### 2.2 In vivo chronic restraint stress model

Following the habituation period, the rats were randomly subdivided into “Control” (*n* = 8 per sex) and “CRS” experimental (*n* = 8 per sex) groups. The Chronic Restraint Stress (CRS) procedure, as described by Smith (2012), was conducted on a benchtop located in a separate room (away from the housing room) to limit the stress inflicted on the Control groups. Each session was conducted between 9:00 and 11:00 a.m. to minimize circadian rhythm effects. The restraint apparatus used included a well‐ventilated and transparent six‐compartment Perspex cage measuring approximately 18 cm × 6 cm × 7 cm. This form of restraint works best for the restraint of mature 10‐week‐old Wistar rats (Smith, 2012). Here, stressed groups were individually restrained for 1 h a day for 28 consecutive days with no access to food or water during the time of restraint. Due to co‐housing in the same room as the Control group, the stressed animals were returned to their cages (after completion of the CRS) but remained in the procedure room for an additional hour of “decompression” to limit the immediate effects of stress pheromones on the Control groups. The Control animals remained in their respective cages in the housing room and were also handled briefly during the restraint period. This omitted the introduction of external stressors and ensured that Control animals remained habituated to handling to avoid triggering acute stress responses during welfare and weekly weighing. A consecutive week of rest followed the 4‐week experimental procedure where no CRS was applied. This ensured that any observed perturbations were more permanent in nature (characteristic of chronic stress) and not masked by acute (more temporary) stress factors. Following the week of rest, rats were moved a short distance from the Tygerberg Animal Facility to the Biomedical Research Institute (Faculty of Medicine and Health Sciences, Stellenbosch University) where they were housed in the Animal Unit for euthanasia. All rats were allowed ~24 h of rest between transport and euthanasia to prevent any influence of the transport on downstream functional/molecular measurements.

### 2.3 Sample collection and preparation

Whole blood samples were collected from all rats for biochemical analyses at several time points throughout the CRS protocol: (a) before the commencement of the experimental period (to serve as the baseline); (b) after the week of rest (post‐CRS); and (c) at the time of death. All blood draws collected during the experimental period were performed under anesthesia (between 7:00 and 9:00 a.m.) via jugular vein bleeding by an experienced animal technician. Euthanasia of the rats occurred individually in a separate room by rapid decapitation using a guillotine to preserve tissue integrity and limit acute stress. Four rats were sacrificed per day with the first euthanasia occurring between 8:00 and 9:00 a.m. and the last between 12:00 and 1:00 p.m. Whole hearts were dissected out, washed with 1× PBS (pH 7.4; P4417; Sigma‐Aldrich), dry‐blotted, and weighed. The whole hearts were then dissected in half in preparation for snap‐freezing using a freeze‐clamp and liquid nitrogen. The frozen tissue was broken into smaller pieces, placed in separate tubes, and stored at −80°C until required for biochemical analyses and respiratory measurements. Samples for oxidative stress assays were prepared in 1:9 ratio with iced 0.1 M PBS (pH 7.4; P4417; Sigma‐Aldrich). Western blotting samples were prepared in 1:9 of non‐denaturing lysis buffer (20 nM Tris–HCl; 1 mM EGTA [pH 7.4]; 1 mM EDTA; 150 mM NaCl; 1 mM β‐glycerolphosphate; 2.5 mM tetrasodium pyrophosphate; 1 mM Na_3_VO_4_; 50 μg/mL PMSF; 0.5% protease inhibitor cocktail [P8340; Sigma‐Aldrich]; 1% Triton X‐100). All samples were homogenized (4°C) using 1.6 mm stainless steel homogenizing beads (1 bead:100 μL lysis buffer) in a bullet blender (2000 × g; three cycles of 1:2‐min runs) to ensure complete subcellular disruption. Homogenized samples were allowed to rest on ice for 15 min before being centrifuged at 12,080 × g for 20 min (4°C). Protein concentration of sample supernatant was quantified using the Bradford protein assay before being aliquoted and storage at −80°C.

### 2.4 Behavior and hormone analyses

Behavioral tests and hormone analyses were performed to validate the effect of the CRS protocol. Briefly, behavioral tests including elevated plus maze (EPM) and tail‐flicks were conducted in accordance with specific protocols as stipulated by Walf and Frye (2007) and Elhabazi et al. (2014), respectively. The EPM test evaluated anxiety‐associated behaviors, while the tail‐flick test assessed acute nociception (Hole & Tjølsen, 2007; Kraeuter et al., 2019). A tail‐flick test was performed on all rats pre‐CRS and served as a baseline measurement, while a final tail‐flick test was performed post‐CRS in addition to an EPM (between 8:00 and 11:00 a.m.) conducted over 2 days. The final tail‐flick test was performed on the first day, followed by the EPM test on the second day. EPM arm interactions provide information on approach‐avoid conflicts and include the time (sec) spent in the respective arms in addition to the number of attempts and entries into open and closed arms (Walf & Frye, 2007). Ethological parameters evaluated risk assessment behavior by the number of performed head‐dips, stretch attempts, and total rears. All behavioral tests were completed in separate novel rooms from the housing and restraint rooms, with tail‐flick procedures being conducted in the same room each time. Data from all behavioral tests was manually recorded for further statistical analyses. Circulating levels of stress hormones, that is, corticosterone and ACTH, were evaluated via ELISA kits (E‐EL‐0160 and E‐EL‐R0048; ElabScience) in stored plasma samples collected from both sex groups at baseline and post‐CRS timepoints. Additionally, trunk blood was collected from female rats post‐CRS at the time of death to determine serum levels of 17β‐estradiol (E_2_; E‐OSEL‐R000; ElabScience).

### 2.5 Oxidative stress

Multiple oxidative stress assays were performed to determine the redox status of the heart tissue. This was achieved by measuring the antioxidant capacity of the samples via an oxygen radical absorbance capacity (ORAC) assay and expressed in Trolox equivalent (TE) units, while the degree of oxidative damage formed by lipid peroxidation was identified using a thiobarbituric acid reactive substance (TBARS) assay. Additionally, the activity of specific antioxidant enzymes such as superoxide dismutase (SOD) and catalase was measured. All samples were loaded in triplicate to minimize the effect of technical errors. A microplate reader was employed to determine absorbance readings for all oxidative stress assays using the microplate data acquisition program (SoftMax® Pro Version 7.0, Molecular Devices, CA, USA). The respective oxidative stress assays were performed at the Applied Microbial and Health Biotechnology Institute at the Cape Peninsula University of Technology (Bellville, Cape Town, South Africa) under the guidance and assistance of an experienced laboratory technician.

### 2.6 Western blotting

The appropriate protein concentrations (12 μL) were prepared with a 3x Laemmli buffer solution (62.5 mM Tris–HCL [pH 6.8]; 4% SDS; 10% Glycerol; 0.03% Bromophenol Blue; and 5% β‐mercaptoethanol) and electrophoresed in various SDS‐PAGE gels. Gels were activated on a UV transilluminator tray using the ChemiDoc™ MP Imaging System and Image Lab™ 6 software (Bio‐Rad Laboratories Inc., Hercules, CA, USA), transferred onto low fluorescence polyvinylidene fluoride membranes (7 min; 2.5 A; 25 V using the Bio‐Rad Trans Blot® Turbo™ Transfer System, Bio‐Rad Laboratories Inc., Hercules, CA, USA), and transfer efficiency assessed before proceeding with blocking. All membranes were blocked for 1 h at room temperature (25°C) with 5% fat‐free milk in TBS‐T solution and incubated in their respective monoclonal primary antibodies (Table 1) overnight (~16 h) at 4°C. Subsequently, the membranes were further incubated at room temperature for an additional hour with the corresponding HRP‐conjugated anti‐mouse (ab97040; Abcam plc., Cambridge, United Kingdom), or anti‐rabbit (#7074; Cell Signaling Inc., Technology, MA, USA,) secondary antibodies. Bands of membranes were detected using Bio‐Rad's Clarity Western Enhanced Chemiluminescent (ECL) substrate (catalog #1705061) and the ChemiDoc™ MP Imaging System (Bio‐Rad, Laboratories Inc., Hercules, CA, USA) and quantified using Image Lab™ 6 software (Bio‐Rad, Laboratories Inc., Hercules, CA, USA). Here, densitometric analysis was performed using total protein as the loading control in Image Lab™ 6 software. Background subtraction was set by using the rolling disc setting. The rolling disc values were set such that the background was subtracted under the band (i.e., peak) of interest in a uniform manner between the lanes of a given blot (Taylor & Posch, 2014). Due to the sample size and loading technique, a total of three gels were required for placement of all samples and repeats. Thus, to account for inter‐gel and loading variability, all samples were normalized to either a normalization control (Nc), containing a “cocktail” of proteins from all samples, or a common control (Cc) sample. The normalized density to this loading control (NDL) for each band was calculated by Image Lab™ software and exported into Excel for further analysis. The NDL for the common control/normalization control (NDLC) of one blot was then divided by the NDLC on another blot, thus providing a normalization factor for each of the blots. The NDL of each sample lane was then multiplied by the respective normalization factors of each blot. All values were then divided by the normalized NDLC to yield a relative fold difference for each sample. These values were then exported into GraphPad Prism 7 (San Diego, CA, USA) for statistical analysis. Data was analyzed in such a manner that all sample protein was represented and expressed as fold change relative to the normalization or common control and expressed in arbitrary units (AU). Proteins that displayed doublet bands were analyzed in unison and represented as a total of the protein of interest (e.g., total Drp1 and total optic atrophy type 1 [OPA1]).

**TABLE 1:** Summary of primary and secondary incubation conditions for Western blotting analysis.

Protein of interest	Size (kDa)	% gel	1° ab	[1° ab]	1° ab diluent	[2° ab]
NDUFB8 (Complex I)	20	12	# 45–8099; Thermo Fisher Scientific; RRID:AB_2533835	1:1000	1% FFM in PBS (0.1 M, pH 7.4)	1:10000
SDHB (Complex II)	30					
UQCRC2 (Complex III)	48					
MTCO1 (Complex IV)	40					
ATP5A (ATP synthase)	55					
OPA1	112	12	# ab119685; Abcam; RRID:AB_10901464	1:1000	5% FFM in TBS‐T	1:10000
PGC‐1α PGC‐1β	92 112	10	# ab188102; Abcam: RRID:AB_3065209	1:1000	TBS‐T	1:1000
Mfn2	86	8	# ab124773; Abcam; RRID:AB_10999860	1:1000	5% FFM in TBS‐T	1:5000
Drp1	83	8	# ab184247; Abcam; RRID:AB_2895215	1:1000	TBS‐T	1:4000

Abbreviations: ATP5A, ATP synthase; FFM, fat‐free milk; MTCO1, mitochondrially encoded cytochrome *c* oxidase I; NDUFB8, NADH dehydrogenase [ubiquinone] 1 beta subcomplex subunit 8; SDHB, succinate dehydrogenase [ubiquinone] iron–sulfur subunit; UQCRC2, cytochrome b‐c1 complex subunit 2.

### 2.7 High‐resolution respirometry

Mitochondrial respiration of frozen heart tissue was assessed using a high‐resolution O2k‐FluoRespirometer (Oxygraph O2k; Oroboros Instruments GmbH, Innsbruck, Austria) following a specialized substrate–uncoupler–inhibitor–titration (SUIT) protocol (SUIT‐004_O2_pfi_DO10) that made use of both complex I (CI)‐ and complex II (CII)‐linked substrates (Doerrier & Gnaiger, 2021). This allowed for the measurement of oxygen consumption, mitochondrial quality, and maximal respiratory ETS capacity in a closed‐chamber system using a Clark‐type oxygen electrode with a high‐resolution and sensitivity to biological samples in the presence of titrated mitochondrial complex substrates and inhibitors (Djafarzadeh & Jakob, 2017). Calibration of the respirometer took place at air saturation (37°C) prior to each experiment. All respiratory data was recorded on a computer at sampling intervals of 2 sec using the DatLab 7.4 O2k acquisition software (Oroboros Instruments GmbH, Innsbruck, Austria). We employed snap‐frozen rat heart tissues that were stored medium‐free for approximately 2–4 months. Approximately 4 mg pieces of tissue of each sample were permeabilized in a digitonin medium (50 μg/mL digitonin in MiR05 kit) on ice for 20 min after physically separating the fibers with tweezers. This permitted the exogenous entry of mitochondrial substrates and the transfer of electrons into the ETS. The tissue fibers were then placed into the two Oroboros chambers containing MiR05 respiration buffer (Gnaiger, 2020). One chamber contained the Control samples while the other contained the CRS samples. The respiration experiments were conducted in parallel in both Oroboros chambers under hyperoxic conditions induced after the addition of the samples and maintained throughout the experiment (maintained ~300–600 μM at 37°C).

Routine respiration was recorded in the absence of exogenous substrates or ADP. This is an indication of the endogenous substrates left in the tissue at the time that they were snap‐frozen and a measure of sample viability. Thereafter, the specific order, volumes, and concentrations of substrates and inhibitors (Sigma‐Aldrich, MO, USA) were titrated into the chambers as detailed in Table 2. Octanoylcarnitine (10 μL, 0.1 M; O6206) and malate (10 μL, 0.05 M; M1000) were added in short succession to induce electron transfer flavoprotein (ETF)‐linked leak respiration. This was followed by ADP (10 μL, 0.5 M; 177,105) to stimulate β‐oxidation‐linked OXPHOS. The addition of cytochrome *c* (5 μL, 4 mM; C7752) allowed for the assessment of outer membrane integrity while the combined addition of pyruvate (5 μL, 2 M; S2378) and glutamate (10 μL, 2 M; G1626) stimulated CI‐linked OXPHOS. Furthermore, CII‐linked OXPHOS was induced by the addition of succinate (20 μL, 1 M; S2378). Multiple titrations of the uncoupling agent carbonyl cyanide 3‐chlorophenylhydrazone (CCCP) (1 μL, 1 mM; C2759) stimulated the maximal ETS capacity. Next, the addition of rotenone (1 μL, 1 mM; R8875) (complex I inhibitor) allowed for the evaluation of CII contribution to this capacity. Shortly after, Antimycin‐A (1 μL, 5 mM; A8674) was titrated to induce residual oxygen consumption by inhibiting complex III (CIII) activity. This provided insight into the leakiness of the mitochondrial membrane and the integrity of the ETS. Complex IV (CIV)‐linked respiration was assessed by the joint addition of ascorbate (5 μL, 0.8 M; A7631) and tetramethyl‐ρ‐phenylenediamine (TMPD; 5 μL, 0.2 M; T3134). Ascorbate functions to maintain TMPD in a reduced state, permitting the artificial supply of electrons from TMPD to complex IV. Lastly, the addition of sodium azide (60 μL, 0.2 M; S2002) inhibited complex IV activity. This was used to identify the true complex IV activity by subtracting the oxygen flux in the presence of sodium azide from that of ascorbate and TMPD. The oxygen flux of all respiratory parameters for each sample was normalized to complex‐IV activity and weight of the tissue to correct for variations in cell mitochondrial content in the oxygraph chambers (Koizumi et al., 2023; Larsen et al., 2012). Refer to Figure S4 for a representative oxygraph.

**TABLE 2:** The SUIT protocol employed using the Oroboros O2k respirometer to determine the mitochondrial respiratory activities of frozen rat heart tissue.

Agent	Volume (μL)	Concentration in titration (M)	Agent type	Site of action	Catalog number
Octanoylcarnitine (*1*)	10	0.10 (in H_2_O)	Substrate	ETF	O6206
Malate (*2*) #	10	0.05 (in H_2_O)	Substrate	CI	M1000
ADP (*3*)	10	0.50 (in H_2_O)	Substrate	ATP synthase	177,105
Cytochrome *c* (*4*)	5	0.004 (in H_2_O)	Substrate	CIV	C7752
Pyruvate (*5*)	5	2.00 (in H_2_O)	Substrate	CI	S2378
Glutamate (*6*) #	10	2.00 (in H_2_O)	Substrate	CI	G1626
Succinate (*7*)	20	1.00 (in H_2_O)	Substrate	CII	S2378
CCCP (*8*)	1	0.001 (in EtOH)	Uncoupler	∆μ_H+_	C2759
Rotenone (*9*)	1	0.001 (in EtOH)	Inhibitor (CI)	CI	R8875
Antimycin‐A (*10*)	1	0.005 (in EtOH)	Inhibitor (CIII)	CIII	A8674
Ascorbate (*11*)	5	0.80 (in H_2_O)	Substrate	CIV	A7631
TMPD (*12*) #	5	0.20 (in H_2_O)	Substrate	CIV	T3134
Sodium azide *(13)*	60	0.20 (in H_2_O)	Inhibitor (CIV)	CIV	S2002

*Note*: Order of substrates/inhibitors indicated in parentheses.

Abbreviations: #, Titrations that directly follow previous agent without line stabilization; ΔμH+, proton gradient; CI, complex I; CII, complex II; CIII, complex III; CIV, complex IV.

### 2.8 Statistical analysis

All data was analyzed using GraphPad Prism 7 (GraphPad Software Inc., San Diego, CA, USA) and expressed as means ± standard deviation (SD). The normality of the data was tested using a Shapiro–Wilk test, while outlier detection made use of the Grubbs test (*α* = 0.05). A Student *t*‐test was conducted where appropriate while a two‐way analysis of variance (ANOVA) was conducted to compare groups. A Fisher's LSD test allowed correction for multiple comparisons for parametric and non‐parametric data. Analyses of mitochondrial respiratory data were performed under the guidance of an expert based at the Centre for Statistical Consultation (Department of Statistics and Actuarial Sciences, Stellenbosch University, South Africa). A statistical value of *p* ≤ 0.05 was accepted as significant across all data sets.

## 3 RESULTS

### 3.1 CRS induced limited changes in percentage weight gain and behavior

Our findings demonstrate that the CRS protocol induced limited changes in percentage weight gain and behavior (Table 3). A nonsignificant change towards reduced percentage weight gain (*p* = 0.06) occurred in stressed males versus Control groups, while females displayed similar weight gain between experimental groups. Behavioral tests revealed no significant alterations in tail‐flick latency between Control versus CRS groups in male or female rats, neither at baseline nor at the post‐CRS timepoint. Exposure to the CRS protocol did not induce changes in the EPM arm interactions or ethological parameters of female rats, while stressed males completed a higher number of closed‐arm attempts (*p* = 0.04).

**TABLE 3:** The effect of CRS on percentage weight gain and behavioral parameters in male and female rats.

Parameter	Male			Female		
	Control	CRS	*p* value	Control	CRS	*p* value
*n*	8	8		8	8	
Anthropometric data						
Weight gain (%)	25.01 ± 5.98	19.70 ± 7.77	0.06	15.24 ± 3.24	14.14 ± 3.85	0.69
Behavioral tail‐flick test						
Baseline latency (sec)	2.77 ± 1.29	3.08 ± 1.97	0.73	4.26 ± 2.11	4.04 ± 1.50	0.80
Post‐CRS latency (sec)	1.97 ± 0.65	2.58 ± 1.25	0.41	2.82 ± 1.99	2.96 ± 1.71	0.85
Behavioral EPM test						
Open‐arm attempts	6.88 ± 2.98	7.13 ± 3.18	0.86	5.13 ± 1.86	6.13 ± 2.64	0.47
Closed‐arm attempts	0.13 ± 0.35	1.13 ± 1.55	0.04*	0.38 ± 0.52	0.50 ± 0.76	0.79
Open‐arm entries	1.38 ± 1.30	1.63 ± 1.69	0.76	2.38 ± 2.20	1.88 ± 1.13	0.54
Closed‐arm entries	5.63 ± 2.00	7.63 ± 1.85	0.06	8.88 ± 1.89	7.75 ± 2.25	0.27
Time in open arms	11.63 ± 12.52	28.38 ± 35.78	0.20	29.00 ± 28.13	27.38 ± 20.11	0.90
Time in closed arms	274.00 ± 14.80	240.90 ± 46.14	0.08	235.60 ± 39.21	233.10 ± 37.00	0.89
Total rears	17.38 ± 5.04	18.25 ± 5.68	0.70	20.50 ± 3.16	18.63 ± 3.38	0.41
Total stretch attempts	2.38 ± 1.30	2.75 ± 1.75	0.65	3.38 ± 1.60	3.38 ± 1.77	>0.99
Total head‐dips	4.00 ± 1.77	6.13 ± 4.26	0.22	8.50 ± 3.42	6.38 ± 3.50	0.22

*Note*: Data presented as means ± SD. Two‐way ANOVA statistical test was performed with Fisher's LSD post hoc correction. Significance values indicate *p ≤ 0.05.

### 3.2 CRS altered circulating estradiol but not stress hormones

**FIGURE 1:**
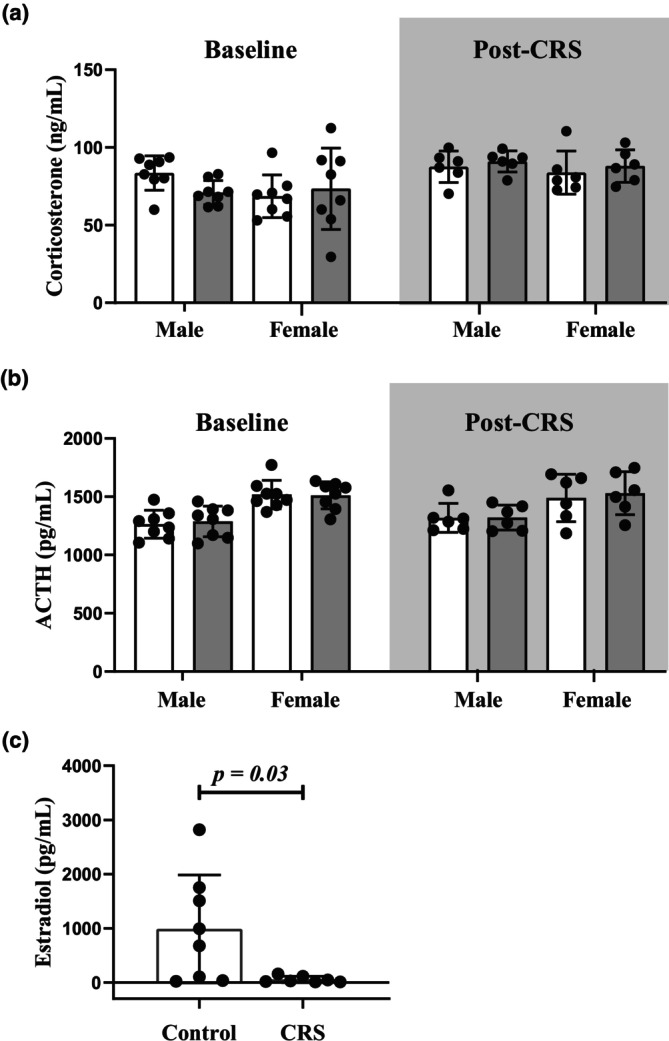
Chronic stress altered circulating female estradiol but not stress hormone levels. (a) No significant differences occurred between Control and CRS rats in plasma corticosterone at both baseline (*n* = 8/group/sex) and post‐CRS (*n* = 6/group/sex); (b) plasma ACTH remained similar between Control and CRS groups at both baseline (*n* = 8/group/sex) and post‐CRS (*n* = 6/group/sex); (c) female serum estradiol levels decreased in CRS (*n* = 7) rats compared to their female Control (*n* = 8) counterparts. Data presented as means ± SD. A Student's *t*‐test (estradiol) or two‐way ANOVA statistical test was performed with Fisher's LSD post hoc correction. Note, although data for (a) and (b) are represented as one figure panel to allow for a single visualization of all findings, statistical analysis for baseline and post‐CRS data were done independently and were not compared. Key: White, Control group; gray, CRS group.

We employed ELISA‐based assays to evaluate plasma levels of ACTH and corticosterone as biological markers of HPA axis activity (Figure 1). Levels of corticosterone remained unchanged between Control and CRS groups at baseline and post‐CRS timepoints for both male and female rats (Figure 1a). Likewise, the CRS model induced no significant changes in ACTH levels of either sex group between the Control and the stress cohorts (Figure 1b). However, our data revealed decreased circulating estradiol (Figure 1c) in stressed females (*p* = 0.03) following CRS compared to their Control counterparts.

### 3.3 CRS and myocardial redox status

**FIGURE 2:**
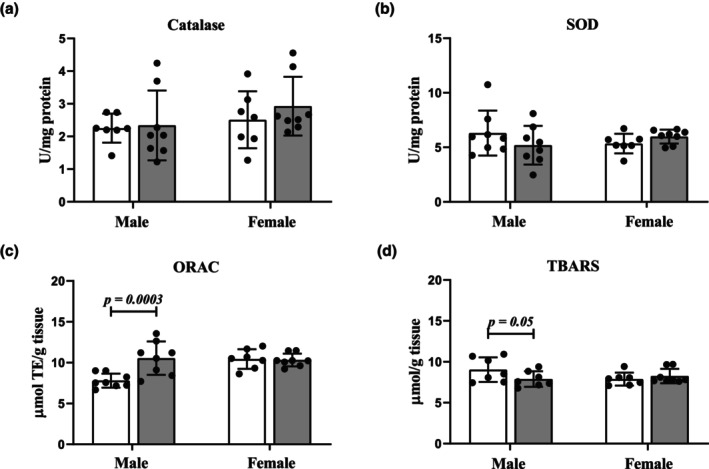
Assessment of myocardial redox status in response to CRS. (a) Catalase activity was unchanged in both stressed male and female hearts (*n* = 8 per sex) compared to the sex‐matched Controls (*n* = 7 per sex); (b) SOD activity remained similar in stressed rats (*n* = 8 per sex) compared to their Control female (*n* = 7) and male (*n* = 8) counterparts; (c) Stressed males displayed an elevation in ORAC versus Controls (*n* = 8 per group) while CRS female hearts (*n* = 8) remained similar to Controls (*n* = 7); (d) TBARS were elevated in stressed males versus Controls (*n* = 7 per group) while no changes occurred between female groups (Control: *n* = 7; CRS: *n* = 8). Data presented as means ± SD. A two‐way ANOVA statistical test was performed with Fisher's LSD post hoc correction. Key: White, Control group; gray, CRS group.

The effects of CRS on the endogenous redox status in the heart were evaluated through enzymatic (catalase and SOD) and nonenzymatic (ORAC) antioxidant capacity levels as well as the presence of lipid oxidative damage using TBARS evaluation. Our data revealed that the CRS model induced sex‐specific oxidative perturbations (Figure 2). No significant changes in catalase (Figure 2a) or SOD (Figure 2b) activity were observed between Control versus CRS for either male or female hearts. However, a prominent increase (*p* = 0.0003) in ORAC (Figure 2c) and a simultaneous decrease in TBARS concentration (*p* = 0.05; Figure 2d) were observed only in male CRS hearts.

### 3.4 CRS‐induced respirometry perturbations in male rat hearts

**FIGURE 3:**
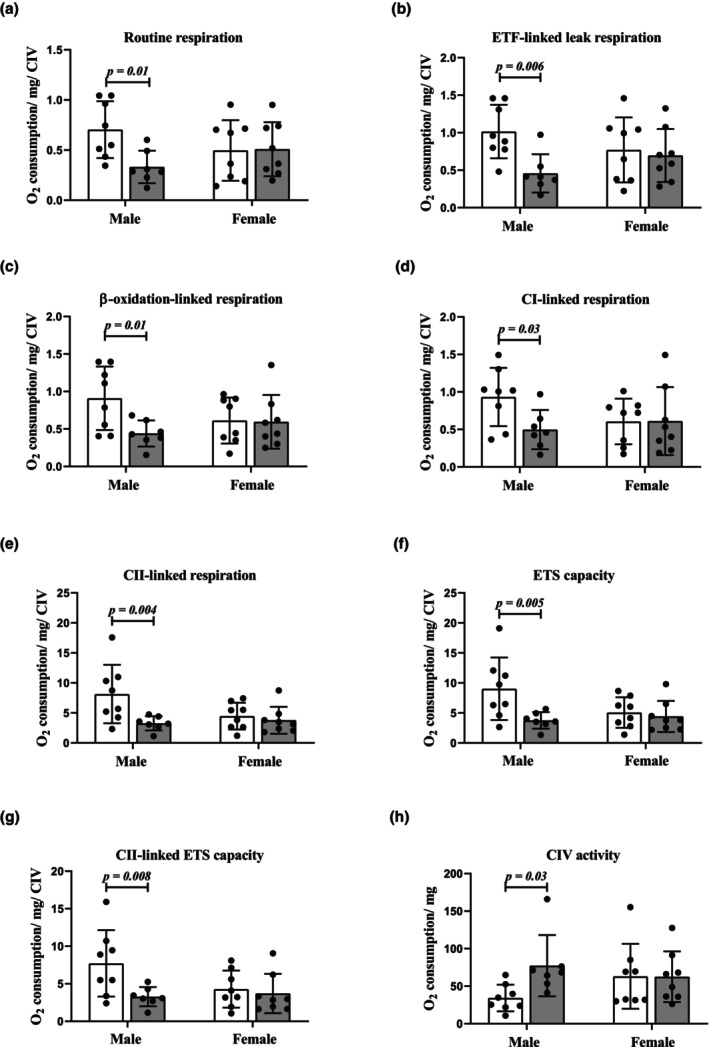
The effects of CRS on mitochondrial oxidative phosphorylation pathways in myocardial tissue. CRS males (unlike females) displayed altered mitochondrial function versus Controls in various respiratory parameters including: (a) Routine respiration; (b) ETF‐linked leak respiration; (c) β‐oxidation‐linked OXPHOS; (d) complex I‐linked OXPHOS; (e) complex II‐linked OXPHOS; (f) ETS capacity; (g) contribution of complex II to ETS capacity; and (h) complex IV activity. Data presented as means ± SD. A two‐way ANOVA statistical test was performed with Fisher's LSD post hoc correction. Key: White, Control group; gray, CRS group; CI, complex I; CII, complex II; CIV, complex IV; white bars, Control group; gray bars, CRS group. Sample size: Male (Control: *n* = 8; CRS: *n* = 7); female (*n* = 8 per group).

High‐resolution respirometry was used to measure the effect of CRS on respiratory functional parameters in snap‐frozen, whole heart tissues stored in the absence of a cryoprotective medium. The ETS integrity, state of coupling, and functionality of such mitochondrial preparations were further confirmed by various flux control and coupling efficiency ratios (Table 4). Additionally, analysis of residual oxygen served as a proxy for oxygen wastage attributed to experimental conditions and intracellular sidechains. Of note, one stressed male sample was removed from all the respiratory analyses due to sample mishandling. Our findings demonstrate prominent sex‐specific mitochondrial respiratory perturbations across several parameters in response to stress (Figure 3). Compared to Controls, male CRS rat hearts demonstrated attenuated oxygen flux related to routine respiration (*p* = 0.01, Figure 3a), ETF‐linked leak respiration (*p* = 0.006, Figure 3b), β‐oxidation‐linked respiration (*p* = 0.01, Figure 3c), complex I‐linked respiration (*p* = 0.03, Figure 3d), complex II‐linked respiration (*p* = 0.004, Figure 3e), ETS capacity (*p* = 0.005, Figure 3f), complex II‐linked ETS capacity (*p* = 0.008, Figure 3g), and an increase in complex IV activity (*p* = 0.03, Figure 3h). Conversely, female CRS rats displayed oxygen flux profiles that were similar to Controls with no alterations detected for any of the respiratory parameters.

**TABLE 4:** The effect of CRS on mitochondrial flux control and coupling efficiency ratios in snap‐frozen rat heart tissue.

	Male			Female		
	Control	CRS	*p* value	Control	CRS	*p* value
Flux control ratios						
R/E	0.09 ± 0.03	0.09 ± 0.02	0.95	0.10 ± 0.03	0.13 ± 0.09	0.17
L/E	0.14 ± 0.08	0.12 ± 0.03	0.55	0.15 ± 0.05	0.17 ± 0.07	0.63
L/P	0.14 ± 0.08	0.12 ± 0.03	0.59	0.15 ± 0.05	0.17 ± 0.06	0.61
P/E	1.01 ± 0.03	1.00 ± 0.03	0.26	1.00 ± 0.02	1.00 ± 0.04	0.64
Coupling efficiency ratios						
PE ratio (P‐L/E)	0.87 ± 0.08	0.88 ± 0.04	0.93	0.85 ± 0.05	0.83 ± 0.05	0.44
EL ratio (E‐L/E)	0.86 ± 0.08	0.88 ± 0.03	0.55	0.85 ± 0.05	0.83 ± 0.07	0.63
PL ratio (P‐L/P)	0.86 ± 0.08	0.85 ± 0.05	0.57	0.88 ± 0.03	0.83 ± 0.06	0.11

*Note*: Data represented as means ± standard deviation. A two‐way ANOVA statistical test was performed with Fisher's LSD post hoc correction. Key: R, routine respiration; L, ETF‐linked leak respiration; E, ETS capacity; P, OXPHOS‐linked respiration.

### 3.5 CRS altered major ETS complex protein levels in a sex‐dependent manner

**FIGURE 4:**
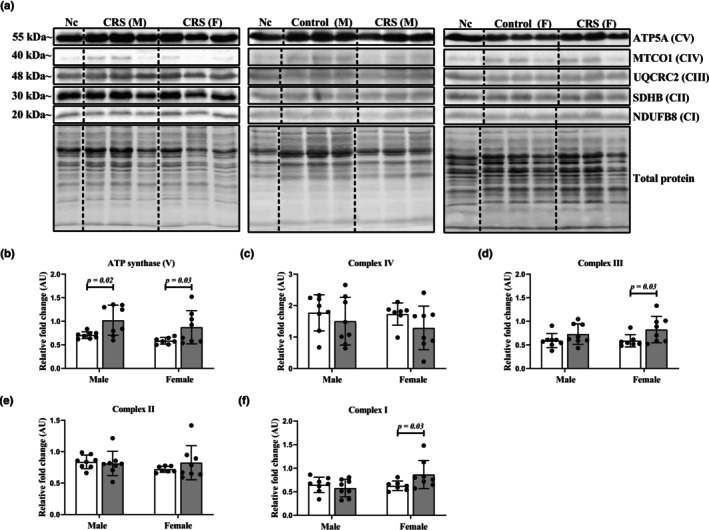
The effects of CRS on mitochondrial OXPHOS protein levels. (a) Representative chemiluminescent image of OXPHOS proteins within myocardial tissue; (b) quantification of protein bands for ATP synthase displayed increases in CRS male and female hearts versus Controls; (c) quantification of protein bands for complex IV demonstrated no significant differences versus Controls for either males or females; (d) complex III protein quantification revealed increases in CRS female myocardial tissue that were absent in CRS males when compared to Controls; (e) quantification of protein bands for complex II demonstrated no significant differences between CRS versus Controls in both sexes; (f) protein levels of complex I were only moderately increased in stressed female hearts when compared to Controls. Data expressed as means ± SD fold change relative to normalization control (Nc). A two‐way ANOVA statistical test was performed with Fisher's LSD post hoc correction. The images for the blotting data were produced from “cutting and pasting” stress and control groups together as they were not loaded next to each other on the original gels. To clearly highlight this, we have included dividing lines as indicated in the figure. Note, the Western blot images used were derived from three singular blots (refer to Figure S1). Separate segments of each blot were represented for each protein of interest to allow for individual visualization of findings. The order in which the segments appear corresponds to the blot from which they were originally derived from. Key: White  Control group; gray, CRS group; M, male; F, female; kDa, kilodaltons; CI, complex I; CII, complex II; CIII, complex III; CIV, complex IV; CV, ATP synthase; NDUFB8, NADH dehydrogenase [ubiquinone] 1 beta subcomplex subunit 8; SDHB, succinate dehydrogenase [ubiquinone] iron–sulfur subunit; UQCRC2, cytochrome b‐c1 complex subunit 2; MTCO1, mitochondrially encoded cytochrome *c* oxidase I; ATP5A, ATP synthase. Sample size: Male (*n* = 8 per group); female (Control: *n* = 7; CRS: *n* = 8).

As mitochondrial OXPHOS is an essential component of energy production, protein expression of ETS complexes I–IV and ATP synthase was assessed to identify stress‐induced effects. Our data show that the CRS protocol increased protein levels of ATP synthase (Figure 4b) in male (*p* = 0.02) and female (*p* = 0.03) stressed hearts, while an increase in complexes III (*p* = 0.03, Figure 4d) and complex I (*p* = 0.03, Figure 4f) were only observed in female heart tissues. Conversely, CRS did not induce changes in complex IV (Figure 4c) and II (Figure 4e) for either sex group.

### 3.6 CRS altered mitochondrial dynamics and biogenesis proteins

**FIGURE 5:**
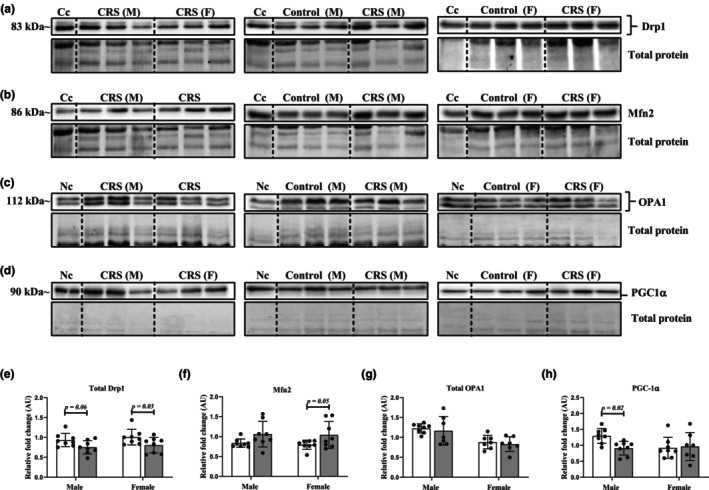
The effects of CRS on mitochondrial dynamic and biogenesis protein levels. (a) Representative chemiluminescent image of Drp1 within myocardial tissue; (b) representative chemiluminescent image of Mfn2 within myocardial tissue; (c) representative chemiluminescent image of OPA1 in myocardial tissue; (d) representative chemiluminescent image of PGC‐1α within myocardial tissue; (e) quantification of protein bands for total Drp1 revealed a sex‐dependent decrease in stressed females that was statistically absent (*p* = 0.06) in stressed males compared to their Control counterparts (*n* = 8/group/sex); (f) protein quantification of Mfn2 revealed increases in CRS females compared to Controls while no changes occurred between male groups (*n* = 8/group/sex); (g) quantification of protein bands for total OPA1 demonstrated no significant differences between Control versus CRS rats for males (Control: *n* = 8; CRS: *n* = 7) or females (*n* = 7 per group); (h) quantification of protein bands for PGC‐1α showcased a moderate decrease in stressed males compared to their Control counterparts (*n* = 8 per group) which was absent in female rats (Control: *n* = 8; CRS: *n* = 7). Data expressed as means ± SD fold change relative to either a normalization control (Nc) that contained protein from each sample or a common control (Cc). Proteins that displayed doublet bands were analyzed in unison as a total of the protein of interest. A two‐way ANOVA statistical test was performed with Fisher's LSD post hoc correction. Note, the images for the blotting data were produced from “cutting and pasting” stress and control groups together as they were not loaded next to each other on the original gels (Figures S2 and S3). To clearly highlight this, we have included dividing lines as indicated in the figure. Key: White, Control group; Gray, CRS group; M, male; F, female; kDa, kilodaltons.

Prominent mitochondrial fission protein, Drp1, and fusion proteins OPA1 and Mfn2 were evaluated to gain insights into the nature of mitochondrial dynamics under chronic stress (Figure 5). Doublet bands observed in Western blotting of Drp1 and OPA1 likely represent post‐translational modifications, splice variants, and/or separate isoforms as previously reported for these proteins (Chen, Gong, et al., 2009; Kamei et al., 2005; Miyai et al., 2019; Olichon et al., 2002; Smirnova et al., 1998; Taguchi et al., 2007). These doublet bands were analyzed in unison and represented as a total of the protein of interest. Analyses of total Drp1 showed a modest decrease in protein levels (*p* = 0.03) in female CRS hearts while male CRS heart tissues displayed a nonsignificant (*p* = 0.06) decrease compared to Controls (Figure 5e). Conversely, analysis of fusion protein levels identified increased Mfn2 levels (*p* = 0.05) in female stressed hearts with no such changes occurring in stressed male hearts compared to Controls (Figure 5f). Analysis of total OPA1 revealed no significant differences between Control versus CRS in protein levels in either males or females (Figure 5g). Additionally, PGC‐1α levels were also assessed to gain further insights into the mitochondrial quality and quantity control systems in stressed hearts. Individual analysis of PGC‐1α revealed a decrease (*p* = 0.02) in male CRS hearts which was absent in CRS females when compared to Controls (Figure 5h).

## 4 DISCUSSION

Although chronic stress is a global health issue that can trigger cardiometabolic diseases, the underlying mechanisms driving such complications remain relatively unclear and warrant further investigation. The main findings of this preclinical study were the following: (i) mitochondrial perturbations represent persistent changes in response to stress and (ii) chronic stress elicited distinct sex‐based differences in mitochondrial respiratory function and regularity proteins.

### 4.1 Mitochondrial perturbations represent persistent changes in response to stress

Mitochondrial respiratory function was assessed via high‐resolution respirometry on snap‐frozen tissue samples stored in the absence of a cryoprotective medium. Although it is known that freezing can affect and lower mitochondrial function, several laboratories found that mitochondrial function and primary changes in mitochondrial activity can be successfully detected in frozen samples, albeit to a lesser degree compared to the gold standard fresh tissue samples (Acin‐Perez et al., 2021; Acin‐Perez et al., 2020; Giovarelli et al., 2023; Krajčová et al., 2020; Osto et al., 2020). Acin‐Perez et al. (2020) identified that mitochondrial respiration can be assessed in freeze‐thawed samples while still reflecting the different experimental interventions. This was confirmed in our analysis of respiratory ratios (Table 4) which demonstrated intact mitochondrial functionality and viable results despite freezing. Additionally, the analysis of flux control and coupling efficiency ratios also revealed that such perturbations—in contrast to previous work—were not due to uncoupling and did not impact coupling control systems (Acin‐Perez et al., 2020; Ebanks et al., 2023). Our results therefore demonstrate that myocardial tissues exhibited consistent coupling efficiency ratios across all experimental groups despite the freezing process.

Unexpectantly, our results revealed that such mitochondrial functional perturbations occurred in the absence of systemic changes in circulating levels of ACTH and corticosterone, while estradiol levels were significantly downregulated. Regarding the relatively limited changes found for ACTH and corticosterone levels, a recent article by Young et al. (2021) sheds potential light on what may be occurring. It is possible that this situation could arise due to an increase in the negative feedback regulation on the HPA axis and/or an attenuated response to CRH at the pituitary level. This may be an attempt by the rats to adapt and cope with chronic psychological stress. Regarding lowered estradiol levels, there is evidence that stress can result in decreased estradiol concentrations. For example, some researchers found that higher stress ratings in women were characterized by lower salivary estradiol concentrations (Roney & Simmons, 2015). The causes of such an inhibition may be mediated by upstream modulators and/or the downstream metabolism of estradiol in the liver. For example, psychological stress can inhibit the hypothalamic–pituitary‐gonadal axis via regulators such as cortisol, CRH, and vasopressin, while downstream, it can increase cytochromes P450 in the liver that would be predicted to increase the breakdown of estradiol into various metabolites such as estrone (Ferin, 1999; Konstandi et al., 2014; Roney & Simmons, 2015).

An increase in closed‐arm attempts identified enhanced risk assessment and hesitancy to explore in stressed males, whereas an increase in open‐arm attempts would be evidential of lower anxiety and/or higher impulsiveness, as seen in previous studies (Riul et al., 2016). Despite this, other EPM parameters and tail‐flick tests revealed no anxiety‐like or nociception behavioral differences in the stressed rats across both sexes (Schneider et al., 2011). These results differ from others who showed downstream mitochondrial dysfunction/alterations in the presence of at least one systemic parameter characteristic of stress (as reviewed by Kaplan et al., 2023). Our data also differ from other studies that demonstrated multiple clear EPM parameter differences in animals exposed to models of chronic stress (Cairns et al., 2024; Nozaki et al., 2020; Salehpour et al., 2019; Shao et al., 2015; Zhvania et al., 2022). However, it is important to note that such behavioral tests have been majorly designed and reported in male rats, that is, considering their social hierarchy and the requirement for males to show aggression/dominance to “earn” the right to impregnate the females, with female rats grossly underrepresented in stress studies. This implies that such behavioral tests likely fall short when comparing males to females. In support, a social‐isolation comparative study between males and females revealed that the EPM may not be the most effective model for female anxiety‐like behaviors and that parameters of this model be tailored to align with female‐specific normal cues (Butler & Weiner, 2016). Such problematic issues will likely influence the interpretation of such behavioral test results in the female rats.

**FIGURE 6:**
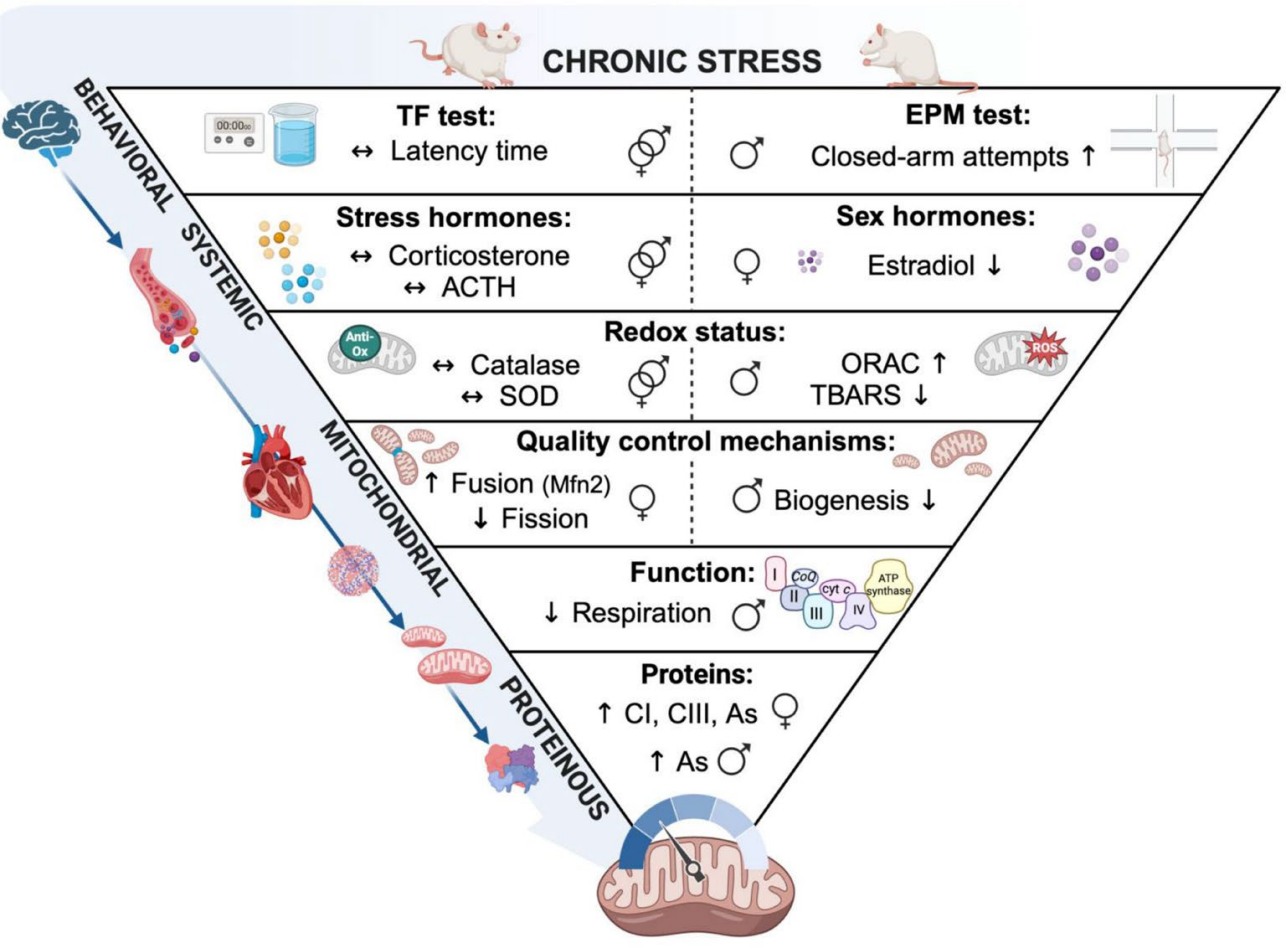
A proposed model of the mitochondrion as a putative gauge for chronic stress detection. Our results demonstrated that mitochondrial functional perturbations occurred in the absence of changes in circulating stress hormones and with limited changes in behavioral parameters. This data suggests that perturbations at the mitochondrial level represent persistent changes in response to chronic stress. Key: ↑, increased; ↓, decreased; ↔, unchanged; CI, complex I; CIII, complex III; As, ATP synthase. The image was created using a licensed version of BioRender.com.

Our results may be suggestive of two other possible phenomena: (i) such cardiac mitochondrial perturbations demonstrate the earliest changes that occur in response to chronic stress or (ii) the rats were relatively efficient in terms of recovery from the chronic stress at a hormonal and behavioral level, although such transient changes lag at the mitochondrial level. The former phenomenon is particularly relevant in conditions of heart pathophysiology where mitochondrial defects manifest during the initial stages of heart disease, acting as an early and potential indicator of heart failure (Lemieux et al., 2011). Regarding the second possibility, it is important to consider that the rats experienced a week of rest with no CRS intervention before the collection of behavioral and stress hormonal data. This may have provided sufficient time for the effects of chronic stress to be mitigated at hormonal and behavioral levels, while mitochondrial perturbations persisted. In agreement, the transient nature of corticosterone and ACTH has been observed in several stress studies (Bowman et al., 2001; Gadek‐Michalska et al., 2013; Hauger et al., 1988; Vahl et al., 2005). Unchanged circulating stress hormonal levels at the end of the experimental period may also indicate some degree of habituation to the CRS protocol. However, this does not exclude the earlier cascade of events triggered by chronic stress that converge on mitochondrial perturbations. Thus, we propose that the mitochondrion may act as a potential sex‐dependent gauge to chronic stress and is sensitive to the cellular changes irrespective of the classical macroscopic effects that are usually expected and observed under conditions of stress (Figure 6).

### 4.2 Chronic stress elicited distinct sex‐based differences in mitochondrial respiratory function and regulatory proteins in frozen tissues

The mitochondrion is particularly influenced by estradiol, which is known to regulate its structure, biogenesis, and activity by increasing ETS efficiency and preventing dysfunction (Chen et al., 2005; Leal et al., 2020; Velarde, 2013). Despite a lack of change in stress behavioral parameters and stress hormones, the stressed females exhibited a sharp decrease in estradiol levels compared to their control counterparts. This agrees with previous literature on animal and human studies, that is, attenuated estrogen and reduced peak levels are commonly reported in response to stress and support the notion that the female rats were indeed stressed (Assad et al., 2017; Roney & Simmons, 2015). As glucocorticoids such as cortisol are known to exhibit inhibitory effects on the hypothalamic–pituitary‐gonadal axis (reviewed by Tilbrook et al., 2009), it is natural to expect that such hormones may be the potential mediator(s) controlling the decreased stress‐induced estradiol levels. However, since this phenomenon occurred in the presence of unchanged corticosterone levels in stressed females, it indicates that other regulatory factors are likely responsible for the decreased estradiol levels observed (Roney & Simmons, 2015). This is supported by research on nonhuman species where the suppressive effects of psychosocial stress on gonadotropins occurred even among adrenalectomized animals or those administered glucocorticoid receptor antagonists (Ferin, 1999; Roney & Simmons, 2015; Tilbrook et al., 2009; Wagenmaker et al., 2009). Despite such lowered estradiol levels, the stressed females appear to be protected and continue to maintain their mitochondrial functionality to a similar extent as their matched controls. In contrast, male rats were more vulnerable to the effects of stress, and this is clearly manifested at multiple levels in terms of mitochondrial function and its control mechanisms.

Mitochondrial functionality and “health” can be assessed by ETS capacity and routine respiration parameters and is often referred to as “mitochondrial reserve capacity” (Brand & Nicholls, 2011). This reserve capacity serves to meet additional energy demands required outside of routine respiration in response to stress to avoid an ATP crisis (Marchetti et al., 2020). Cardiac tissues of stressed males displayed decreased respiration in these two parameters which may be indicative of damage to the ETS and that may result in a limited electron flow, hampered oxygen consumption, decreased ATP generation, and overall diminished respiration. This aligns well with other studies performed on male mice where chronic stress functionally impaired mitochondrial OXPHOS, decreased efficiency of oxygen utilization, and attenuated complex I‐driven respiration in various brain regions (Gong et al., 2011; Kambe & Miyata, 2015). Furthermore, a recent study demonstrated a significantly reduced ETS capacity of isolated mitochondria in the skeletal muscle of male mice subjected to chronic variable stress (Nikolic et al., 2023). This inhibition of the ETS followed by a diminished reserve capacity is a common feature of heart diseases under conditions of severe cardiac stress (Chacko et al., 2014; Desler et al., 2012; Gong et al., 2003; Kingsley‐Hickman et al., 1990). Additionally, low mitochondrial function and reserve capacity may be characteristic of metabolic stress and indicative of decreased substrate availability or metabolic disruption induced by chronic stress as observed in previous studies (Chacko et al., 2014; Gao et al., 2014; Li et al., 2010; Wang et al., 2009). In these studies, substrate intermediates (most notable fatty acid levels and succinate) associated with Krebs cycle‐linked metabolic pathways were considerably lowered in stress groups compared to the controls, indicative of a dysfunctional Krebs cycle. This is particularly relevant for cell types such as cardiomyocytes that largely depend on fatty acids as a major energy fuel substrate. Additionally, the omnivorous nature of the heart allows for a shift in resilience to more energy‐efficient fuels (e.g., glucose) with a decrease in fatty acid β‐oxidation as an adaptive mechanism, particularly in cardiac pathology and dysfunction (Lopaschuk et al., 1994; Michalak & Agellon, 2018; Ritterhoff et al., 2020; Tran & Wang, 2019; Wende et al., 2017; Wu et al., 2022). However, our data revealed that stressed male hearts displayed decreased respiration via both fatty acid‐ and glucose oxidation‐linked pathways. Such impaired fuel substrate points to a global attenuation of fuel substrate utilization and that is not necessarily a substrate‐specific phenomenon. Stressed male rats also exhibited an increase in complex IV oxygen consumption rate and ATP synthase enzymatic content. Complex IV and ATP synthase represent vital regulatory centers of OXPHOS and are ATP demand dependent. Complex IV is regulated by the binding of various effectors including ATP‐mediated feedback inhibition (Kadenbach, 2021). A study by Ramzan et al., (2010) proposed that an increase in the ADP:ATP ratio results in the mitigation of an allosteric ATP‐inhibition of complex IV in frozen bovine heart mitochondria. It is therefore reasonable to assume that an increase in complex IV respiration and ATP synthase protein content may be due to an abolished feedback inhibition and indicative of a compensatory mechanism in response to the chronic stress‐induced attenuation of cellular energy production and substrate availability/supply (Desler et al., 2012; Kadenbach, 2021; Villani & Attardi, 2000). We therefore argue that this, together with decreased ETS capacity and routine respiration, likely represents a global sex‐specific shift of cardiac mitochondrial respiration in male rat hearts in favor of an energy‐sparing effect in response to chronic stress due to upstream metabolic dysfunction.

In contrast to males, stressed females preserved cardiac mitochondrial respiration and displayed no major deviations for any of the respirometry parameters when compared to controls. Further investigation into OXPHOS protein levels revealed a significant increase in complex I, III, and ATP synthase content in response to stress. This is expected as the role of mitochondrial sexual dimorphisms and cardioprotective effects in females is well described in the heart (Colom, Oliver, et al., 2007; Ventura‐Clapier et al., 2017). Similar results were obtained in a caloric restriction study where it was postulated that the higher OXPHOS enzyme activities (complex I, III, and ATP synthase) in the skeletal muscle of female rats could be explained by an enhanced ability of females to adapt to altered metabolic energy conditions (Colom, Alcolea, et al., 2007). Although it is well documented that estrogens such as 17β‐estradiol can upregulate the expression of mitochondrial DNA (mtDNA) genes to encode ETS complex proteins to elicit direct and indirect effects on ETS activity and confer cardioprotective effects, the upregulation of these proteins occurred concurrently with lowered estradiol levels (Chen et al., 2005; Chen, Cammarata, et al., 2009; Mendelsohn, 2000; Yang et al., 2004). In contrast to our findings, previous work demonstrated that lowered estradiol attenuated the expression of cardiac mitochondrial complex I, IV, and ATP synthase (Guajardo‐Correa et al., 2022; Pavón et al., 2017). In our study, despite the substantial reduction in estradiol in stressed females and the statistical increases in the protein expression of complexes I, III, and ATP synthase, we did not observe any changes in mitochondrial respiration. This is suggestive that reduced estradiol may have elicited a compensatory increase in the expression of the mitochondrial protein complexes to maintain the same level of mitochondrial function as controls.

Mitochondria can adapt to stressful intrinsic or extrinsic conditions and preserve their function and viability by promoting either a fragmented or fused phenotype (Liu et al., 2020). Our results revealed decreased protein levels of myocardial Drp1 in addition to increased Mfn2 levels in stressed female rats, while OPA1 expression levels remained unaltered in both stressed males and females compared to the controls. This suggests that the CRS model we established was relatively mild in severity and elicited a sex‐dependent increase in fusion as opposed to fission mechanisms in stressed female hearts. This is characteristic of mitochondrial adaptation versus maladaptation in response to chronic stress (Adaniya et al., 2019; Chen et al., 2011, 2012; Jheng et al., 2012). We propose that such attenuated fission protects female cardiac mitochondria from degradation via mitophagy to preserve energy production and safeguard overall mitochondrial health. Moreover, increased fusion promotes the formation of larger and more interconnected mitochondria that allows for mitochondrial complementation and the augmentation of energy production to offer protection against mitochondrial damage. Conversely, stressed male hearts failed to upregulate fusion and decrease fission, contributing to the reduction in ETS performance. Previous studies have also linked mitochondrial dynamics to the balance between energy and nutrient supply, implying that alterations in mitochondrial morphology may function as a mechanism for bioenergetic adaptation during cardiac pathological remodeling (Vásquez‐Trincado et al., 2016). This agrees with our notion of attenuated substrate supply/availability (possibly due to Krebs cycle dysfunction) as the cause for decreased mitochondrial respiration in stressed male rats, while the role of increased fusion and a higher bioenergetic efficiency protects stressed female hearts from the manifestation of such perturbations at a mitochondrial respiratory level. Although estradiol preserves mitochondrial function and confers benefits during times of stress by promoting Mfn2‐mediated fusion in the cardiovascular system and attenuating mitochondrial fission, the decreased estradiol levels demonstrated in our stressed females suggest that an unidentified mechanism is likely responsible for such cardioprotective dynamic changes (Beikoghli Kalkhoran & Kararigas, 2022; Mooga et al., 2018). However, it is important to take into consideration that the proteins orchestrating mitochondrial dynamics are also regulated by post‐translational modifications as well as protein interactions. These regulating factors require further investigation to fully understand the threshold concepts substantiating the process of altered mitochondrial dynamics in response to chronic stress.

Metabolic regulatory events that dictate the fuel selection and capacity of ATP production are also regulated at the gene expression level (Huss & Kelly, 2005). Fatty acid oxidation rates and mitochondrial respiratory function are maintained under normal conditions via the interaction of PGC‐1α with other transcription factors (Chen et al., 2022; Huss & Kelly, 2005). This interaction drives the regulation of enzymes involved in fatty acid uptake and enhances the expression of fatty acid β‐oxidation, OXPHOS, and ETS enzymes/complexes to collectively regulate downstream cardiac mitochondrial oxidative capacity, rates of fatty acid β‐oxidation, coupled respiration, and energy expenditure (Chen et al., 2022; Gleyzer et al., 2005; Huss & Kelly, 2005; Lehman et al., 2000; Oka et al., 2020; Panagia et al., 2005; Russell et al., 2004; Schreiber et al., 2003; Vega et al., 2023). As we found a sex‐dependent decrease in PGC‐1α protein levels, we propose that this change together with the concurrent decrease in mitochondrial respiration and the absence of increased respiratory complex levels supports a relatively energy‐deficient state in stressed male hearts (Oka et al., 2020). Our results are consistent with several animal studies that highlight cardiac metabolic and downstream mitochondrial respiratory defects in PGC‐1α knockout mice, such as a blunted expression of OXPHOS genes, reduced oxidation of both fatty acids and glucose pathways, and repressed OXPHOS capacity (Arany et al., 2005; Chen et al., 2022; Kärkkäinen et al., 2019). In the context of stress, our findings are in line with Zhan et al., (2018) where mice subjected to a chronic model of social defeat exhibited decreased PGC‐1α expression levels at multi‐organ levels. Furthermore, a recent sex‐specific study of chronic social stress exhibited a higher decline in cardiac stress resistance linked with repressed mitochondrial biogenesis in male mice compared to their female counterparts (Helman et al., 2022). Such studies support our findings of decreased PGC‐1α in male rats and their increased susceptibility to chronic stress. Conversely, females generally demonstrate increased resilience to stress via the upregulation/expression of PGC‐1α, primarily enabled by estrogen effects (Witt et al., 2008). We also found that the females maintained respiratory function and upregulated ETS‐associated protein content in the absence of increased PGC‐1α. It appears that female rats retain such resilience even at relatively low circulating estrogen levels.

As the heart is a highly metabolic organ with extensive energy demands and limited regenerative capabilities, it relies heavily on antioxidant defenses for protection against oxidative stress (Geddie et al., 2023). Exposure to chronic stress induced no changes in the enzymatic antioxidant activity of SOD or catalase in both sex groups, while stressed male hearts exhibited an elevation in the nonenzymatic antioxidant capacity with decreased lipid peroxidation. This data is in agreement with previous work by our group, where a model of chronic unpredictable mild stress failed to produce any significant changes in SOD and catalase of stressed male rat hearts but induced a significant elevation in antioxidant capacity (Geddie et al., 2023). In contrast to our study, this model of unpredictable mild stress induced a mild oxidative phenotype with elevated lipid peroxidation that may indicate a model‐dependent influence of chronic stress on cardiac redox systems. As SOD functions as the first line of defense to catabolize superoxide into hydrogen peroxide (which is neutralized into water by catalase) it is reasonable to assume that the downstream quantity of ROS produced in response to our model of chronic stress was insufficient to elicit changes in the activities of the antioxidants measured. Despite the neutralizing power of SOD on superoxide, the resulting hydrogen peroxide is still reactive and can promote cellular damage (Fujii et al., 2022). However, a decrease in lipid peroxidation and increased antioxidant capacity in stressed male hearts may suggest that ROS production and the subsequent antioxidant scavenging may be occurring via an alternative redox route than those measured in this study. As an increase in enzyme activity generally indicates an adaptive response to combat oxidative stress, our results suggest that the male cardiac total antioxidant capacity was elevated in response to chronic stress and rising oxidative stress and that this adaptive mechanism was efficient in attenuating pro‐oxidant load (Alkadhi, 2013; Geddie et al., 2023). This compensatory reliance on antioxidant defense in stressed male hearts may partially be attributed to the incapacity to increase Mfn2 and the failure to instill a more protective fusion phenotype, as observed in the female hearts, where redox integrity was well maintained. Other studies investigating sex‐linked differences in cardiomyocytes found that female rats were more protected against oxidative damage, generating less ROS with higher detoxification mechanisms than males, even after insults such as ischemia/reperfusion injury and caloric restriction (Colom, Oliver, et al., 2007; Lagranha et al., 2010). Therefore, the absence of redox changes in response to chronic stress may be due to such cardioprotective effects in females. Additionally, as a well‐known antioxidant, the decrease in estradiol may account for the absence of upregulated antioxidant capacity (represented by ORAC) in response to the CRS protocol. However, this limitation was absent in males who are not subjected to such fluctuations in estradiol. Such results provide insight into the sex‐dependent nature of the heart under chronic stress and underscore its efficiency in counteracting stress‐induced oxidative damage.

## 5 LIMITATIONS AND FUTURE RECOMMENDATIONS

We acknowledge that a limitation of this study was the lack of significant stress‐induced changes in stress hormones (i.e., corticosterone and ACTH) and the behavioral parameters measured. This may be due to the week of rest that may have caused some degree of re‐adaptation after the stress period. Due to sample limitations, we were unable to investigate blood chemistry to support heart dynamics, to test whether there would be an upregulation of glycolytic enzymes, and to also evaluate mitochondrial protein activity based on housekeeping proteins that account for mitochondrial mass at both Western blotting (e.g., voltage‐dependent anion channels [VDAC] and/or mitochondrial heat shock protein 70) and mitochondrial respiratory levels (citrate synthase). In terms of the latter, this would more accurately reflect alterations in mitochondrial protein activity with the mitigation of any other additional influences related to mitochondrial density. Furthermore, the selection of cardiac tissue for molecular analysis and high‐resolution respirometry was conducted at random as we did not consider the different anatomical regions of the heart. This should be an important consideration for future research work as the heart consists of several anatomical regions containing different mitochondrial subpopulations that respire at varying levels (Hollander et al., 2014; Kuznetsov & Margreiter, 2009). Finally, in our summary diagram (Figure 6) we propose that oxidative stress may be implicated in such pathological processes. However, it should be noted that our oxidative stress assays were completed on whole cells and not using isolated mitochondria, meaning that there may also be extra‐mitochondrial sources of ROS at play in this instance.

We recommend that further investigations be conducted in a sex‐dependent manner but should focus on upstream regulators of mitochondrial biogenesis (e.g., citrate synthase), cardiac metabolism (e.g., Krebs cycle intermediates), and the activation of mitochondrial fission‐fusion proteins to better determine the downstream effects of chronic stress and the influence of sexual dimorphisms on such effects. We also advocate that future studies should address compartment‐specific activities of antioxidants such as cytosolic SOD.

## 6 CONCLUSION

Despite behavioral tests and stress hormone levels showing limited changes in stressed rats compared to their control counterparts, the mitochondrion emerges as a key cellular target of chronic stress. Our data revealed several mitochondrial perturbations in stressed males, whereas females exhibited a more protected and resilient phenotype despite lowered estradiol levels. Together, we postulate that our results hint that the mitochondrion may act as a potential sex‐dependent gauge in response to chronic stress (Figure 6). However, further molecular studies are required to explore this postulate and understand these underlying mechanisms driving sex‐specific responses and the effects of chronic stress on mitochondrial function and clinical outcomes.

## AUTHOR CONTRIBUTIONS

Caitlin Paige Odendaal‐Gambrell, Cassidy O'Brien, Megan Cairns, and M. Faadiel Essop conceived and designed the study. Caitlin Paige Odendaal‐Gambrell, Cassidy O'Brien, and Fanie Rautenbach performed the experiments. Caitlin Paige Odendaal‐Gambrell, Cassidy O'Brien, Gerald J. Maarman, Danzil E. Joseph, Megan Cairns, Carine Smith, Fanie Rautenbach, Jeanine L. Marnewick, and M. Faadiel Essop analyzed the data and prepared the figures. Caitlin Paige Odendaal‐Gambrell, Cassidy O'Brien, Gerald J. Maarman, and M. Faadiel Essop interpreted the results. Caitlin Paige Odendaal‐Gambrell, M. Faadiel Essop, and drafted, edited, and revised the manuscript. All authors have read and approved the final version of the manuscript.

## FUNDING INFORMATION

The authors wish to acknowledge funding provided by the National Research Foundation (NRF) of South Africa to M. F. Essop (grant numbers: 129325 and CPRR230508103517).

## CONFLICT OF INTEREST STATEMENT

The authors declare that the research was conducted in the absence of any commercial or financial relationships that could be construed as a potential conflict of interest.

## ETHICS STATEMENT

Ethical clearance was granted by the Animal Care and Use Research Ethics Committee of Stellenbosch University (#ACU‐2022‐19,400), and all handling and treatment of the rats aligned with the accepted standards for the use of animals in research and teaching as reflected in the South African National Standards 10,386:2008.

## Supporting information


Figure S1.

Figure S2.

Figure S3.

Figure S4.

## Data Availability

The data that support the findings of this study are available on request from the corresponding author and will be openly available in a repository when the article is accepted for publication.
