# Supplementary material for: Chronic stress elicits sex‐specific mitochondrial respiratory functional changes in the rat heart
Source: Physiol Rep. 2025 May 12;13(9):e70371. doi: 10.14814/phy2.70371 (PMC12069860; doi:10.14814/phy2.70371)
Supplement: Supplementary file 1 — Figure S1. [file PHY2-13-e70371-s002.pdf]

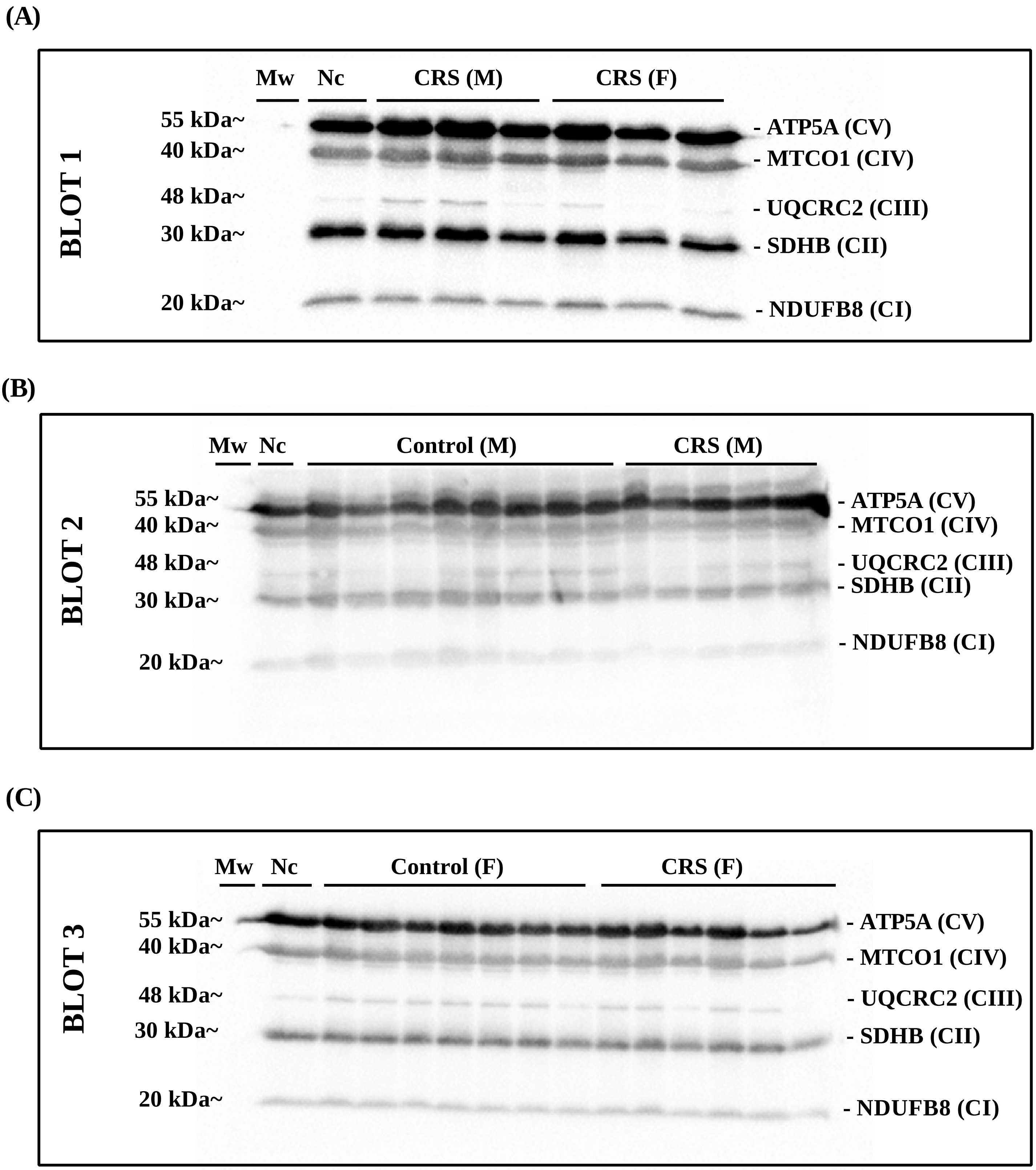

Supplementary Figure 1 Whole representative chemiluminescent images of mitochondrial OXPHOS proteins complex I-V within myocardial tissue across three blots.
