# Supplementary material for: Chronic stress elicits sex‐specific mitochondrial respiratory functional changes in the rat heart
Source: Physiol Rep. 2025 May 12;13(9):e70371. doi: 10.14814/phy2.70371 (PMC12069860; doi:10.14814/phy2.70371)
Supplement: Supplementary file 2 — Figure S2. [file PHY2-13-e70371-s004.pdf]

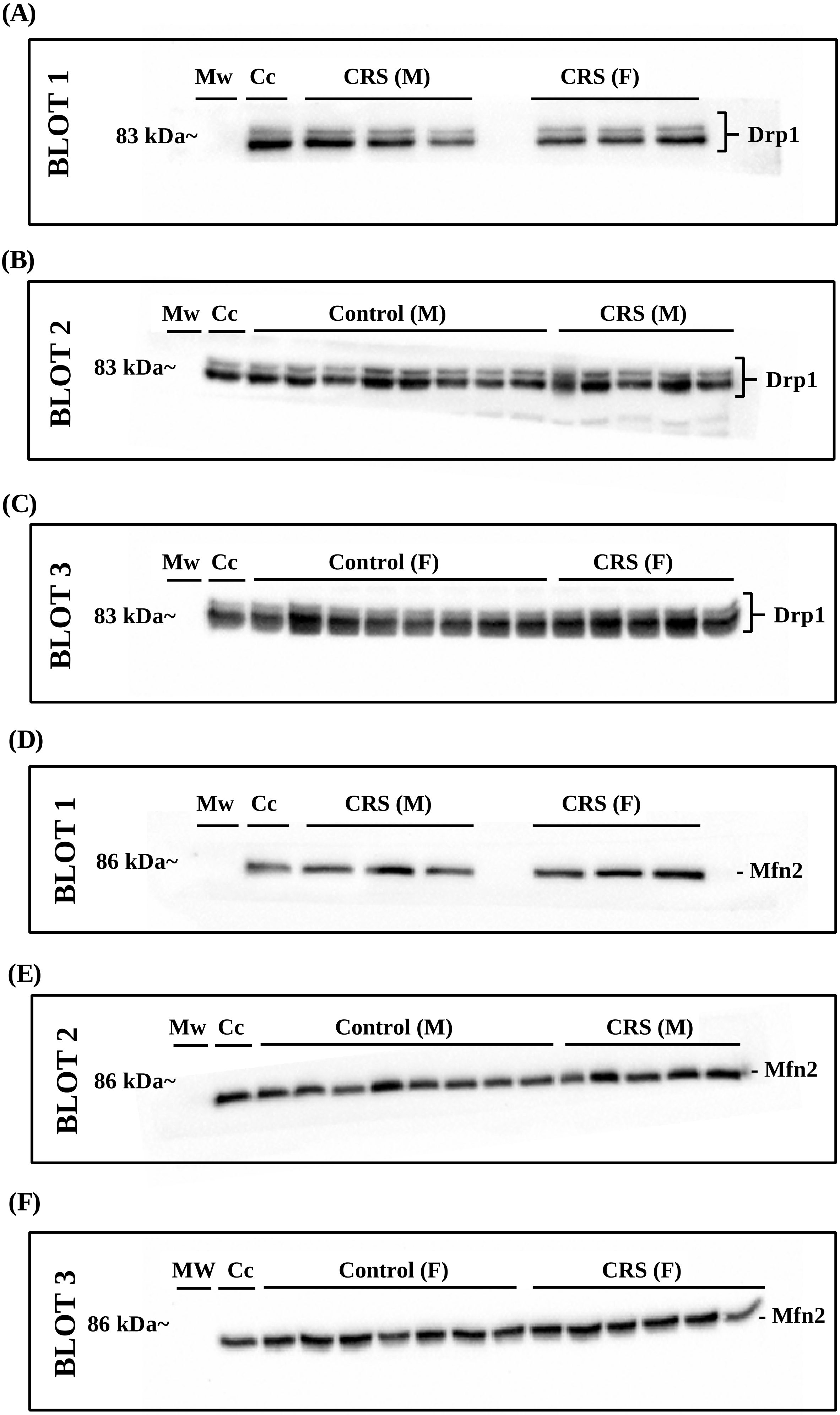

Supplementary Figure 2 Whole representative chemiluminescent images of mitochondrial Drp1 and Mfn2 protein within myocardial tissue across three blots.
