# Supplementary material for: Chronic stress elicits sex‐specific mitochondrial respiratory functional changes in the rat heart
Source: Physiol Rep. 2025 May 12;13(9):e70371. doi: 10.14814/phy2.70371 (PMC12069860; doi:10.14814/phy2.70371)
Supplement: Supplementary file 4 — Figure S4. [file PHY2-13-e70371-s003.pdf]

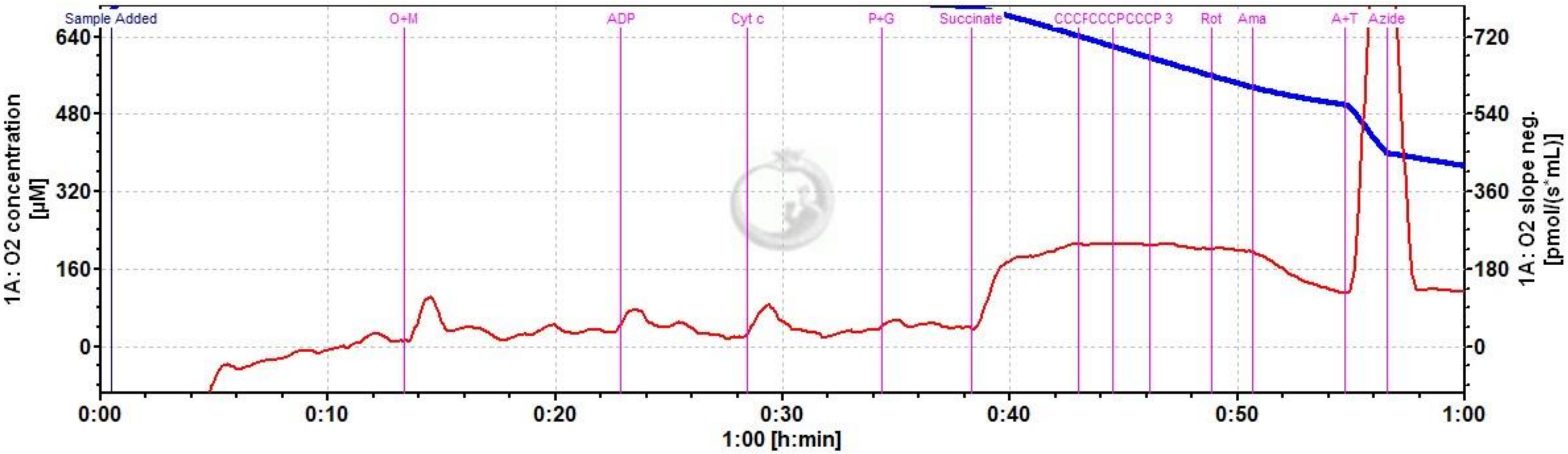

Supplementary Figure 4 A representative oxygraph trace measuring OXPHOS in permeabilized frozen myocardial tissue indicating oxygen (O2) concentration (μM) and specific O2 flux [pmol/(s*mL)].
